# Commutativity of probabilistic belief revision

**DOI:** 10.3389/fcogn.2025.1623227

**Published:** 2025-08-06

**Authors:** Bart Jacobs

**Affiliations:** iHub, Radboud University, Nijmegen, Netherlands

**Keywords:** Bayesian updating, multiset, commutativity, notation, cognition

## Abstract

Bayesian updating, also known as belief revision or conditioning, is a core mechanism of probability theory, and of AI. The human mind is very sensitive to the order in which it is being “primed”, but Bayesian updating works commutatively: the order of the evidence does not matter. Thus, there is a mismatch. This paper develops Bayesian updating as an explicit operation on (discrete) probability distributions, so that the commutativity of Bayesian updating can be clearly formulated and made explicit in several examples. The commutativity mismatch is underexplored, but plays a fundamental role, for instance in the move to quantum cognition.

## 1 Introduction

In mathematics an operation is called commutative if swapping its arguments does not change the outcome, as in addition of numbers: *n*+*m* = *m*+*n*. Also, actions can be called commutative when the effect does not depend on the order in which they are taken: if I first give someone *n* Euros and then another *m* Euros, the financial effect is the same as first giving you *m* and then *n* Euros. However, the emotional effect on the receiver's side may be quite different, for instance when *n* is much greater than *m*: first giving the higher amount *n* then *m* may lead to disappointment, whereas after first giving the lower amount *m* and then *n* the receiver may end up in a more positive mood.

Similar differences are well-known in human cognition, especially when one looks at how the mind processes (new) information—in what is called priming. The order of such priming (or updating) is highly relevant. Here is a simple example, involving two sentences *p* and *q*, about Alice and Bob, which will be presented below in two orders: (a) as *p* and *q*, and (b) as *q* and *p*. This makes no difference in (Boolean) logic, but watch carefully what effect the different orders has on your understanding of the situation.

(a) “Alice is sick” and “Bob visits Alice”;
(b) “Bob visits Alice” and “Alice is sick.”

In the first case (a) you may think that Bob is a nice guy, but maybe less so in the second case (b). It is surprising how quickly the human mind makes a (causal) connection. The strength of this effect depends on many factors, including one's own background (priors). Check for instance what the effect is of replacing in the above two sentences “sick” with “pregnant.” This dependence on the order is called the order effect in Uzan (2023). It is the main motivation to switch to quantum logic in cognition theory (see e.g., Busemeyer and Bruza, 2012; Yearsley and Busemeyer, 2016; Gentili, 2021), since conjunction (“and”) in quantum logic is not commutative.

This paper offers reflections on commutativity in probability theory, and in particular in probabilistic updating. Bayesian updating is introduced as an explicit operation that takes a probability distribution ω with some form of evidence *p* and produces a new, updated distribution ω|*p*. This formulation generalizes the common approach. The (mathematical) details will appear later, but at this stage it is relevant to emphasize that this new formulation of Bayesian updating makes it possible to clearly express commutativity: for two pieces of evidence *p* and *q* one has:


(1) $$\omega {|}_{p}{|}_{q}=\omega {|}_{q}{|}_{p}.$$

In words: updating a distribution ω first with *p* and then with *q* gives the same result as updating ω first with *q* and then with *p*. This commutativity cannot be expressed using the traditional notation *P*(*E*∣*D*) of conditional probabilities, since it leaves the distribution implicit.

To add some terminology: this updating is also called conditioning or belief revision. The distribution ω, before the update, is called the prior, and the distribution ω|*p*, after the update, is called the posterior. The task of computing the posterior distribution is called inference, or also (probabilistic) learning.

Probabilistic updating is an essential part of the ongoing AI-revolution, in various forms, via learning and training. Generative and conversational AI are becoming part of professional and private environments. If these tools are meant to behave like humans, their updating should be non-commutative, as illustrated above, with the different effects resulting from different orders (a) and (b). Bayesian updating, however, is commutative (Equation 1). Hence there is a mismatch [as also emphasized in Uzan (2023)]. One aim of this paper is increase the awareness of this gap, by making the commutativity of Bayesian updating explicit, in a new form (Equation 1).

The paper starts with some simple observations about lists and multisets. One can have a list of letters, say (*a, b, c, a, c, b, a*). In a list, elements can occur multiple times and the order of their occurrence matters. Notice that in a subset like {*a, c*}, elements can occur at most once, and their order does not matter. Mulitsets are “inbetween” lists and subsets: elements may occur multiple times, but their order does not matter. The latter property makes them relevant in the current context. Multisets are a highly undervalued datatype. The fact that they are so little used may be part of our poor understanding of the role of commutativity. We can already make a connection with what we saw above: updating a distribution with two pieces of evidence—as in Equation 1—should not be done with a list (*p, q*) or (*q, p*), but with a multiset of evidence containing both *p* and *q*, where the order does not matter. Section 2 below starts with an informal introduction to multisets and ends with some notation and definitions that are relevant in this setting.

Multisets form a natural preparation for (discrete probability) distributions, in Section 3. Distributions keep count of elements via probabilities or weights (in the unit interval [0, 1]) that add up to one. Multisets can be turned into “fractional” distributions via normalization. A basic fact is that every distribution can be obtained as limit of such fractional distributions, just like every real number can be obtained as a limit of fractions. Mathematically, this is described as: the set D(X) of distributions on a finite set *X* is a compact complete metric space, with (normalized) multisets as dense subset, see Theorem 1. This denseness formalizes the “frequentist” perspective on probability distributions, as results of long-term experiments.

Section 4 introduces Bayesian updating ω|*p* in concrete form, shows that traditional notation *P*(*E*∣*D*) is a special case, and illustrates usage of updates ω|*p* in two examples. The first one is a rather straightforward application, where a prior bird distribution is updated after a bird count. If there is another bird count one year later, the bird distribution can be updated once again. It turns out that eventhough the counts are chronologically ordered, this order is irrelevant for the distribution updates. The second example is more challenging. It involves various inference questions about the sex and ages of children in a family (with a twin), after specific observations. The possibilities for the offspring are represented as multisets, based on Section 2. The prior distribution in this case is a distribution over these multisets. Again, the order of the multiple updates does not matter. This basic fact is proven in general form at the end of this section, see Proposition 1.

The commutativity of Bayesian updating may be seen as folklore knowledge but it is hardly made explicit in the literature. One reason is that the standard formulation of conditional probabilities *P*(*E*∣*D*) does not lend it self to a commutativity result, as above in Equation 1, since it hides the distribution, assuming there is only one implicit distribution. Hence one cannot express facts about different distributions via traditional notation. Our formulation of Bayesian updating ω|*p* as an operation on distributions thus has advantages—as hopefully also becomes clear from the illustrations in Section 4. The Appendix derives the update formulation ω|*p* from the traditional formulation via Kadison duality. This is a new result. The derivation is mathematically sophisticated and is not necessary for the main line of the paper. This line is part of a new approach to probability theory using the language and methods of category theory. In the body of the paper the role of category theory remains in the background and no prior knowledge of that field is required.

Thus, this paper's contributions lie in putting the spotlight on the commutativity of Bayesian updating, in a new form (Equation 1), via a new formulation ω|*p* of this update mechanism, which is both given in concrete form and derived from a fundamental duality result. At the same time, the paper provides a gentle introduction to a new approach to the area, in which multisets and explicitly written distributions play a central role.

## 2 Multisets, with multiplicities of elements

Suppose you check how much money you have in your pocket and you find that you have three 2-Euro coins 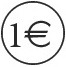 and two 1-Euro coins 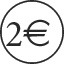. How would you describe this handful of coins mathematically? It is not a subset of coins {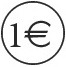, 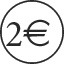}, since such a subset ignores the multiplicities of the coins that you have. One can describe the contents of your pocket as a list, for instance as, (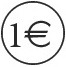, 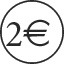, 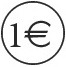, 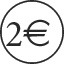, 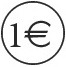), when you lay them out in your hand. But the order of this list is arbitrary and does not reflect your answer: I have three of 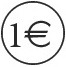 and two of 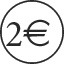.

The proper way to capture the situation mathematically is via a *multiset*. It can be understood as a subset in which elements may occur multiple times, or as a list in which the order does not matter. Unfortunately, there is no established notation for multisets. We use kets |−〉, where we put the elements of the multiset inside the ket and their multiplicity in front. Thus, the coins in your pocket, are properly described as multiset:


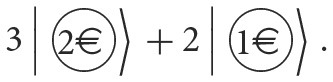


These kets |−〉 are borrowed from quantum theory. They have no mathematical meaning here and are used only to separate the elements of a multiset from their multiplicities.

As a practical example, consider the outcome of an election, say between two candidates Alice (*A*) and Bob (*B*). The outcome may be written as a multiset 55|A〉+45|B〉, indicating that 55 votes are for Alice and 45 votes for Bob. Describing the outcome of the election as a list of length 100, say with the chronological order of casted votes, is a bad idea, for two reasons: the list does not immediately tell what the outcome is, and the list may leak information about who voted for whom—when the order of the voters is recorded.

In which ways can you break a note of 10 Euro into coins of 2 and 1 Euros? The six options can be described as multisets:


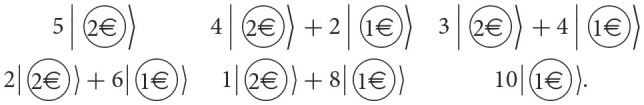


When we are interested in the ways to break the note of 10, we do not care about the order of the coins. When we do describe the break-up options as lists we end up with 10 different lists of coins.

Multisets are a useful “datatype,” in the language of computer science, for keeping counts of elements. However, multisets are often not recognized or expounded as such. For instance, in mathematics, the solutions of a polynomial form a multiset, and not a set, since solutions may occur multiple times. For example, the multiset of solutions of the polynomial *x*^3^−7*x*^2^+16*x*−12 = (*x*−2)(*x*−2)(*x*−3) takes the form 2|2〉+1|3〉, since the number 2 occurs twice as solution and 3 once. Similarly, the eigenvalues of a matrix form a multiset. In the notation 2|2〉+1|3〉 the kets play a useful role, since they make clear which numbers are in the multiset and which numbers are the corresponding multiplicities.

Consider the following basic question. A friend of mine has three children, but I don't know if they are girls (*G*) or boys (*B*). How many offspring options are there? Many people will quickly say: *eight*, namely:


(2) $$\begin{array}{l} G,G,G\;G,G,B\;G,B,G\;B,G,G\\ G,B,B\;B,G,B\;B,B,G\;B,B,B. \end{array}$$

One can also say: there are *four* options, namely with three, two, one, or zero girls. These four options are described as multisets:


(3) 3|G〉 2|G〉+1|B〉 1|G〉+2|B〉 3|B〉.

The eight list options in Equation 2 only make sense if there is an order—*e.g*. by ascending or descending age—but no order was specified in the question. Hence the multiset answer, with four options, seems most natural. It abstracts away from any ordering of the children.

**Figure 1:**
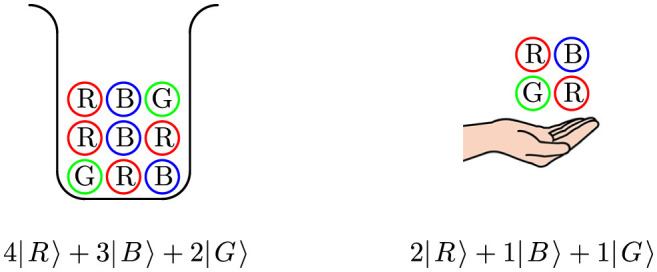
An urn filled with colored balls on the left, written as multiset, and a possible draw from this urn on the right, also written as a multiset. One can ask what is the probability of this draw from the urn. This depends on the mode of drawing: draw-and-delete, draw-and-replace, draw-and-duplicate (see Jacobs, 2022, 2025) for details.

In the next section we illustrate how multisets form the basis for discrete probability theory. Indeed, probabilities naturally arise from counting, possibly in a limit process. Urns filled with balls of different colors form a basic model in probability theory (see e.g. Johnson and Kotz, 1977; Mahmoud, 2008; Ross, 2018) and many other references. Here, such an urn is identified with a multiset over the set of colors (see Figure 1). The multiplicities of the different colors determine the probability of drawing a ball of a particular color. For instance, in this case, the probability of drawing a single red ball is $\frac{4}{9}$. A general draw from such an urn is also a multiset, as on the right in Figure 1.

These introductory observations illustrate that multisets are useful mathematical abstractions for keeping count of multiple occurrences of different objects (or data items). We have mentioned (Equation 1) that the order of data in Bayesian updating is irrelevant. This implies that data in Bayesian learning is best organized as a multiset. Indeed, a histogram of data—with heights in natural numbers—is another example of a multiset.

The next definition fixes the notation and terminology that we shall use in the sequel. In this setting, a multiset involves only finitely many elements from a given set. The multiplicities are natural numbers. One could also allow non-negative real numbers as multiplicities, but we don't need such generalizations. Here and in the sequel we shall use the sign := for definitions.

Definition 1. Let *X* be an arbitrary set.

1. A *multiset* over *X* is a finite formal sum of the form:

$$\begin{array}{cc} n_{1}|x_{1}\rangle +\cdots +n_{k}|x_{k}\rangle & \text{with }\{\begin{array}{l} \text{multiplicities }n_{1},\ldots ,n_{k}\in \mathbb{N}\\ \text{elements }x_{1},\ldots ,x_{k}\in X. \end{array} \end{array}$$

Alternatively, a multiset over *X* may be described as a function φ:*X* → ℕ with finite support *supp*(φ): = {*x* ∈ *X*|φ(*x*) ≠ 0}.
2. The *size* ||φ||∈ℕ of a multiset φ is its total number of elements, including multiplicities. Explicitly, both in ket and function notation:

$$\begin{array}{r} \left\|\sum_{i}n_{i}|x_{i}\rangle \right\|:\text{​}=\sum_{i}n_{i}\text{ and }\left\|\varphi \right\|:=\sum_{x\in supp(\varphi )}\varphi (x)\\ =\sum_{x\in X}\varphi (x). \end{array}$$

3. We shall write M(X) for the set of all multisets over *X*. This operation M is functorial: it works not only on sets *X* but also on functions *f*:*X*→*Y*, and then yields a function M(f):M(X)→M(Y) given by:

(4) $$M\begin{array}{c} \left(f)(\sum_{i}n_{i}|x_{i}\rangle \right):=\sum_{i}n_{i}|f(x_{i})\rangle . \end{array}$$

Notice that in this definition the set *X* may be infinite, but a multiset over *X* has only finitely many elements from *X*, in its support. We freely switch between the ket and function notation for multisets and use whichever is most convenient in a particular situation.

The ket notation involves a formal sum, with some conventions: (1) terms 0|*x*〉 with multiplicity zero may be omitted; (2) a sum *n*|*x*〉+*m*|*x*〉 is the same as (*n*+*m*)|*x*〉; (3) the order and any round brackets in a sum do not matter. Thus, for instance, there is an equality of multisets:


2|a〉+(5|b〉+0|c〉)+4|b〉=9|b〉+2|a〉

These conventions are especially relevant for functoriality in item 3, since multiple elements *x, x*′ may be mapped to the same outcome *f*(*x*) = *f*(*x*′). We use this functoriality especially for projection functions π_*i*_:*X*_1_×*X*_2_→*X*_*i*_. It then yields marginalisaiton.

As briefly discussed in the introduction, it is illuminating to compare multisets to the datatypes of lists and subsets.

**Table:** 

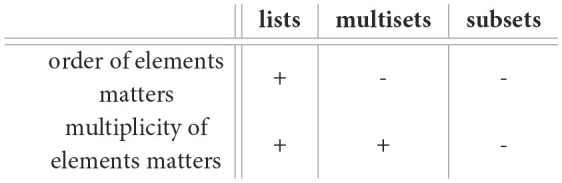

If we write L(X) and P(X) for the sets of finite lists and of subsets over a set *X*, then we can form a diagram in which multisets sit inbetween lists and subsets:


(5) $$L(X)\;\underset{\text{forget order}}{\overset{acc}{\to }}M(X)\;\underset{\text{forget multiplicity}}{\overset{supp}{\to }}P(X)$$

The function *acc* performs accumulation, via *acc*(*x*_1_, …, *x*_*n*_): = 1|*x*_1_〉+⋯+1|*x*_*n*_〉. It counts the occurrences of elements in a list, for instance in:


acc(c,b,a,a,a,b,c)=3|a〉+2|b〉+2|c〉

Similarly, the accumulations of the lists of children in Equation 2 yields the multisets of children in Equation 3.

The support map *supp* in Equation 5 sends a multiset on a set to its subset of elements, see Definition 1 1. In the example, *supp*(3|*a*〉 + 2|*b*〉 + 2|*c*〉) = {*a, b, c*}. As an aside for the mathematically oriented reader, both *acc* and *supp* preserve the monoid structures on these data types and they both are maps of monads. They are fundamental, well-behaved mappings.

## 3 Distributions, with probabilities of elements

This section introduces finite discrete probability distributions, using ket notation as for multisets. It is shown that multisets give rise to “fractional” distributions, via normalization, and that each distribution is in fact a limit of such fractional distributions.

A coin has two sides, namely head (*H*) and tails (*T*). A fair coin assigns a probability of $\frac{1}{2}$ to both sides. In ket notation we write it as on the left below.


$$\frac{1}{2}|H\rangle +\frac{1}{2}|T\rangle \;\frac{51}{100}|H\rangle +\frac{49}{100}|T\rangle .$$

On the right, above, there is an almost fair coin, with a slight bias toward head. Characteristically for distributions, the numbers before the kets must be probabilties from the unit interval [0, 1] that add up to one.

Consider the urn multiset υ = 4|*R*〉+3|*B*〉+2|*G*〉 from Figure 1, with size ||υ|| = 9. The probability of drawing a red ball is $\frac{4}{9}=\frac{\upsilon \left(R\right)}{\left||\upsilon |\right|}$. These draw probabilities arise via normalization of the urn-as-multiset, for which we use a function *flrn*, as in:


$$\begin{array}{l} flrn(\upsilon )=\frac{\upsilon (R)}{\left\|\upsilon \right\|}|R\rangle +\frac{\upsilon (B)}{\left\|\upsilon \right\|}|B\rangle +\frac{\upsilon (G)}{\left\|\upsilon \right\|}|G\rangle \\ \;=\frac{4}{9}|R\rangle +\frac{3}{9}|B\rangle +\frac{2}{9}|G\rangle \end{array}$$

The latter distribution captures the probabilities of drawing a ball of a particular color from the urn υ. The function *flrn* will be defined in general form below. It turns a (non-empty) multiset into a distribution. It learns this distribution by counting, so *flrn* is used as abbreviation of frequentist learning.

As argued in Gigerenzer and Hoffrage (1995), people are in general not very good at probabilistic (esp. Bayesian) reasoning, but they fare better at reasoning with what are called “frequency formats” but which are in fact multisets. The information that there is a 0.04 probability of getting a disease can be captured in a distribution $\frac{1}{25}D\rangle +\frac{24}{25}D^{⊥}$, where *D*^⊥^ stands for no-disease. This appears more difficult to grasp than the information that 4 out of 100 people get the disease, as captured by the multiset 4|*D*〉+96|*D*⊥〉. Applying frequentist learning *flrn* to the latter multiset gives the disease distribution.

Definition 2. Let *X, Y* be arbitrary sets.

1. A *distribution* on *X* is a formal finite convex sum *r*_1_|*x*_1_〉+⋯+*r*_*n*_|*x*_*n*_〉 with elements *x*_*i*_ ∈ *X* and associated probabilities *r*_*i*_ ∈ [0, 1] satisfying $\underset{i}{\sum }r_{i}=1$.
Alternatively, a distribution is given by a “probability mass” function ω:*X* → [0, 1]⊆ℝ with finite support *supp*(ω) = {*x* ∈ *X*|ω(*x*) ≠ 0} and with $\underset{x}{\sum }\omega \left(x\right)=1$.
Each non-empty multiset $\varphi =\sum_{i}n_{i}|x_{i}\rangle \in M(X)$ of size $n=\underset{i}{\sum }n_{i}=||\varphi ||>0$ gives rise to a “fractional” distribution flrn(φ)∈D(X), given by:

$$flrn(\sum_{i}n_{i}|x_{i}\rangle ):=\sum_{i}\frac{n_{i}}{n}|x_{i}\rangle \text{ i.e. }flrn(\varphi ):=\sum_{x\in X}\frac{\varphi (x)}{\left\|\varphi \right\|}x\rangle .\;$$

We write D(X) for the set of distributions on *X*. This D is functorial, like M, see Definition 1 (3): for a function *f*:*X*→*Y* we write D(f):D(X)→D(Y) for the function that produces the image distribution, as:

(6) $$D(f)(\sum_{i}r_{i}|x_{i}\rangle ):=\sum_{i}r_{i}|f(x_{i}).$$

We apply the same conventions to distributions as formal sums of ket's as for multisets, so that terms 0|*x*〉 may be ommitted, *etc*.

Such fractional distributions *flrn*(φ) have fractions as probabilities. We recall that the subset ℚ↪ℝ is dense: each real number can be expressed as limit of fractions. An analogous situation applies to distributions. In order to formulate it we use the following *total variation distance* on distributions. It is a special case of the Kantorovich-Wasserstein distance (Kantorovich and Rubinshtein, 1958). For two distributions $\omega ,\omega^{\prime }\in D\left(X\right)$ one defines the distance *d*(ω, ω′)∈[0, 1] as:


(7) $$\begin{array}{ccc} d(\omega ,\omega^{\prime }) & := & \frac{1}{2}\sum_{x\in X}|\omega (x)-\omega^{\prime }(x)|. \end{array}$$

We can now formulate some basic topological properties of distributions. Stated informally, all distributions come from multisets.

Theorem 1. For a finite set *X*, the set D(X) of distributions on *X*, with total variation distance (Equation 7), is a compact complete metric space, containing a countable dense subset of fractional distributions, given as image of frequentist learning *flrn*, from non-empty multisets to distributions.

There is another topic that we need to introduce, namely random variables, together with their expected value—described here as validity.

Definition 3. Let *X* be an arbitrary set.

1. An *observable* on *X* is a function *p*:*X* → ℝ. These observables are closed under pointwise sum + and multiplication &. There are the always-zero and always-one observables **0**, **1**:*X* → ℝ.
We write *Obs*(*X*): = ℝ^*X*^ for the vector space of observables on *X*.
2. A *random variable* is a pair (ω, *p*) of a distribution ω∈D(X) and an observable *p*:*X* → ℝ, on the same set *X*.
3. For a random variable ω∈D(X),p:X→ℝ the *expected value* is written as validity ω⊧*p* and defined as:

(8) $$\begin{array}{l} \begin{array}{ccc} \omega ⊧p & := & \underset{x\in X}{\sum }\omega \left(x\right)\cdot p\left(x\right); \end{array} \end{array}$$

The expected value is commonly written as 𝔼(*p*) with the distribution ω left implicit. This may be inconvenient and confusing, especially when the distribution at hand may change, for instance in a computational setting. The notation 𝔼_*x*←ω_*p*(*x*) fares better since it makes the distribution ω explicit, but it introduces an additional bound variable, namely the *x* that is sampled from ω. However, since the actual probability ω(*x*) of the sampled element *x* does not occur in this expression 𝔼_*x*←ω_*p*(*x*), it can not be used for calculations. Hence we prefer the (new) validity notation $\omega ⊧p=\underset{x}{\sum }\omega \left(x\right)\cdot p\left(x\right)$ for the expected value of observable *p* in distribution ω.

## 4 Bayesian updating

There are two main schools in statistics, one of “frequentist” nature, assigning probabilities to data, and one of “Bayesian” kind, where probabilities are associated with hypotheses. The frequentistist approach is captured, in the discrete case, by distributions $\sum_{i}r_{i}|x_{i}\rangle \in D(X)$, with probabilities *r*_*i*_ associated with elements / objects / data item *x*_*i*_∈*X*. The Bayesian approach may be formalized in terms of belief functions *Obs*(*X*) → ℝ, assigning values to observables / predicates / hypotheses / evidence. In Bayesian approaches these assignments are often called subjective, resulting from individual choices about how much value (like money) people wish to put on which possible outcomes.

The Appendix explores a mathematical perspective and describes an isomorphism (on the left in Equation 13, Appendix) that connects these Bayesian and frequentist approaches via a duality isomorphism HomObs(X),ℝ≅D(X) between belief functions and distributions. Thus, one could say, the matter is solved, there is mathematically no difference between the two approaches—up to isomorphism.

Conditional probabilities are typically developed on the Bayesian side, in terms of adapted belief functions. Using the approach of the Appendix, this approach can be pulled across the duality isomorphism, to the frequentist side. It leads to a form of updating in terms of adapted distributions ω|_*p*_, see Section B in Appendix for the mathematical details. The next definition already formulates Bayesian updating of distributions with observables, in concrete form. It has been developed and used in a series of papers (Jacobs and Zanasi, 2016, 2017; Jacobs, 2017a; Cho and Jacobs, 2019; Jacobs, 2019, 2021, 2024) aimed at systematizing probabilistic updating. It is with this formalization of Bayesian updating that we can clearly formulate and prove commutativity of updating, see Proposition 1 below.

Definition 4. Let ω∈D(X) be a distribution with a non-negative observable *p*:*X* → ℝ≥0 such that the validity ω⊧*p* is non-zero. In that case we define the *Bayesian update* ω|p∈D(X) of ω with “evidence” *p* as the normalized product:


(9) $$\begin{array}{ccc} \omega {|}_{p} & := & \sum_{x\in X}\frac{\omega (x)\cdot p(x)}{\omega ⊨p}|x\rangle . \end{array}$$

This formulation of Bayesian updating comes alive in illustrations. We present an example first and then show how the above formulation ω|*p* generalizes the traditional formulation *P*(*E*∣*D*).

Example 1. We consider a study involving four common species of birds: robin (*R*), crow (*C*), sparrow (*S*), and woodpecker (*W*). We start from the following species distribution (in a particular area), on the set *X* = {*R, C, S, W*}.


$$\begin{array}{l} \sigma \;=\;\frac{1}{4}|R\rangle +\frac{1}{3}|C\rangle +\frac{1}{4}S+\frac{1}{6}|W\rangle \\ \;\approx \;0.25|R\rangle +0.333|C\rangle +0.25|S\rangle +0.167|W\rangle . \end{array}$$

This is our prior distribution. Then a day of bird counting happens, resulting in a count observable *f*:*X* → ℕ with numbers:


$$\begin{array}{ccccccccccccccc} f\left(R\right) & = & 200 & \; & f\left(C\right) & = & 150 & \; & f\left(S\right) & = & 50 & \; & f\left(W\right) & = & 100. \end{array}$$

We see that this observable does not really match the prior, for instance since the number of observed robins is higher than the number of crows, whereas the robin probability in σ is lower than the crow probability. Also, the number of observed sparrows is low with respect to the woodpecker number. Hence we expect that updating σ with *f* will lead to a considerable change of (relative) probabilities.

We first calculate the expected value, as validity:


$$\begin{array}{l} \sigma ⊨f\;\overset{\left(8\right)}{=}\;\sum_{x\in X}\sigma (x)\cdot f(x)\\ \;=\;\sigma (R)\cdot f(R)+\sigma (C)\cdot f(C)+\sigma (S)\cdot f(S)+\sigma (W)\cdot f(W)\\ \;=\;\frac{1}{4}\cdot 200+\frac{1}{3}\cdot 150+\frac{1}{4}\cdot 50+\frac{1}{6}\cdot 100\\ \;=\;\frac{775}{6}. \end{array}$$

(8)

The updated, posterior distribution can now be computed, with this validity as normalization factor:


$$\begin{array}{l} \sigma {|}_{f}\;\overset{\left(9\right)}{=}\;\sum_{x\in X}\frac{\sigma (x)\cdot f(x)}{\sigma ⊨f}|x\rangle \\ \;=\;\frac{1/4\cdot 200}{775/6}|R\rangle +\frac{1/3\cdot 150}{775/6}|C\rangle +\frac{1/4\cdot 50}{775/6}|S\rangle +\frac{1/6\cdot 100}{775/6}|W\rangle \\ \;=\;\frac{12}{31}|R\rangle +\frac{12}{31}|C\rangle +\frac{3}{31}S+\frac{4}{31}|W\rangle \\ \;\approx \;0.387|R\rangle +0.387|C\rangle +0.0968|S\rangle +0.129|W\rangle . \end{array}$$

(9)

This posterior distribution σ|*f* incorporates the evidence of the observable *f*. Its robin and crow probabilities are equal, and its woodpecker probability is higher than the sparrow probability, reflecting the count outcome.

A year later a new bird count happens, resulting in a new observable *g*:*X* → ℕ, say with *g*(*R*) = 100, *g*(*C*) = 150, *g*(*S*) = 100, and *g*(*W*) = 50. This count is more in line with the prior. One can then update σ|*f* once again, now with observable *g*—last year's posterior is this year's prior. The resulting second update takes the form:


$$\begin{array}{l} \sigma {|}_{f}{|}_{g}\;=\;\frac{12}{35}|R\rangle +\frac{18}{35}|C\rangle +\frac{3}{35}|S\rangle +\frac{2}{35}|W\rangle \\ \;\approx \;0.343|R\rangle +0.514|C\rangle +0.0857|S\rangle +0.0571|W\rangle . \end{array}$$

This second update brings us a bit closer to the original prior σ.

Interestingly, this double update σ|*f*|*g* is equal to the update σ|*g*|*f* with swapped observables *f, g*. Thus, eventhough there is a clear order in the yearly bird counting, the mathematics of Bayesian updating ignores this order and produces the same outcome for both orders (*f, g*) and (*g, f*) of observables.

We now show how the conditional probability in traditional form fits into our form of Bayesian updating (9).

Lemma 1. Let ω∈D(X) be a distribution, with a subset (event) *E*⊆*X*. We write **1***E*:*X* → ℝ for the observable given by the indicator function of *E*, with associated validity:


1E(x)    :=   {1if x∈E0if x∈E           and  P(E) :=  ω⊨1E  =  ∑x∈Eω(x).

For another subset *D*⊆*X* one has:

1. **1***E&***1***D* = **1***E*∩*D*;
The conditional probability *P*(*E*∣*D*) is obtained as validity of **1***E* in the distribution ω updated with **1***D*, that is, as:

$$\begin{array}{ccccccc} \omega |1D⊧1E & = & \frac{\omega ⊧1E\&1D}{\omega ⊧1D} & = & \frac{P\left(E\cap D\right)}{P\left(D\right)} & =: & P\left(E|D\right). \end{array}$$

This last equation defines the conditional probability *P*(*E*∣*D*).

**Proof**. 1. For *x*∈*X*, using that & is given by pointwise multiplication:


$$\begin{array}{l} (1_{E}\&1_{D})(x)=1\;\Leftrightarrow \;1_{E}(x)\cdot 1_{D}(x)=1\\ \;\Leftrightarrow \;1_{E}(x)=1\text{ and }1_{D}(x)=1\\ \;\Leftrightarrow \;x\in E\text{ and }x\in D\\ \;\Leftrightarrow \;x\in E\cap D\Leftrightarrow 1_{E\cap D}(x)=1. \end{array}$$

(9)

2. We only have to prove the first equation, since the second one follows from the previous item. Thus:

$$\begin{array}{l} \omega |1_{D}⊨1_{E}\;\overset{\left(9\right)}{=}\;\sum_{x\in X}\omega {|}_{1_{D}}(x)\cdot 1_{E}(x)\\ \;\overset{\left(9\right)}{=}\;\sum_{x\in X}\frac{\omega (x)\cdot 1_{D}(x)}{\omega ⊨D}\cdot 1_{E}(x)\\ \;=\;\frac{\sum_{x\in X}\omega (x)\cdot (1_{D}\&1_{E})(x)}{P(D)}\\ \;=\;\frac{P(E\cap D)}{\left(D\right)}. \end{array}$$

The traditional *P*(−) notation leaves the distribution implicit, which has many disadvantages. Most relevant in this context is that this *P*(−) notation makes it impossible to express the commutativity of Bayesian updating, as formulated in Proposition 1 below.

We include another illustration that combines several of the topics that we discussed earlier: multisets, functoriality (for marginalization), and updating.

Example 2. Consider the following situation and questions, describing a typical update situation with observations about offspring.^1^

A friend of mine has three children aged 4 and 5 with one twin.

(a) What is the probability that there are three girls, assuming that the probability of a girl is $\frac{1}{2}$? 2.
(b) I ring this friend's doorbell and I hear a girl's voice say that she will open the door soon. If I can assume that this is one of the three children, what is the probability that my friend has three daughters? 3.
(c) A 4-year-old boy opens the door. Still assuming this is one of the children, what is the probability that there are three boys?

Questions (b) and (c) are independent.

We address this situation in terms of multisets of children, as in Equation 3. The situation is more complicated now since we have to use a set *C* = {*B, G*} for the (sex of the) children but also a set *A* = {4, 5} for their ages. The possible offspring configurations are (certain) multisets of size 3 over the product set *C*×*A*. After a moment's thought we see that the prior υ is a distribution of the following form.


(10) $$\begin{array}{l} \upsilon =\frac{1}{16}|2|B,4\rangle +1|B,5\rangle \rangle +\frac{1}{16}|1|B,4\rangle +2|B,5\rangle \rangle \\ \;+\;\frac{1}{8}|1|B,4\rangle +1|B,5\rangle +1|G,4\rangle \rangle +\frac{1}{16}|2|B,5\rangle +1|G,4\rangle \rangle \\ \;+\;\frac{1}{16}|1|B,5\rangle +2|G,4\rangle \rangle +\frac{1}{8}|1|B,4\rangle +1|B,5\rangle +1|G,5\rangle \rangle \\ \;+\frac{1}{16}|2|B,4\rangle +1|G,5\rangle \rangle +\frac{1}{8}|1|B,4\rangle +1|G,4\rangle +1|G,5\rangle \rangle \\ \;+\frac{1}{16}|2|G,4\rangle +1|G,5\rangle \rangle +\frac{1}{8}|1|B,5\rangle +1|G,4\rangle +1|G,5\rangle \rangle \\ \;+\;\frac{1}{16}|1|B,4\rangle +2|G,5\rangle \rangle +\frac{1}{16}|1|G,4\rangle +2|G,5\rangle \rangle . \end{array}$$

This υ is a distribution over multisets, using nested kets. The outer, big kets are for the probabilities, with inside the different offspring configurations in the form of a multiset. For instance, the multisets 1|*B*, 4〉 + 2|*G*, 5〉 and 1|*G*, 4〉 + 2|*G*, 5〉 in the last line capture the situations with one boy (or girl) of 4 and two girls of 5 years old.^2^

We shall write *S*: = *supp*(υ) for the support of this distribution υ. This set *S* contains all of the 12 different multisets φ inside the big kets in Equation 10.

For the first question (a) we ask ourselves more generally what the children distribution is in this situation. It can be obtained by discarding the ages, via the first marginal of the multisets inside the big kets. This involves applying the marginalization function $M\left(\pi_{1}\right):M\left(C\times A\right)\to M\left(C\right)$ to these multisets, see Definition 1 3. Since we wish to apply this marginalization function $M\left(\pi_{1}\right)$ inside the bigkets, we have to use functoriality of D as well, see Definition 2 3. Thus, the distribution of children marginals is obtained as:


$$\begin{array}{l} D(M(\pi_{1}))(\upsilon )=\sum_{\varphi \in S}\upsilon (\varphi )|M(\pi_{1})(\varphi )\rangle =\sum_{\varphi \in S}\upsilon (\varphi )|\sum_{x,y}\varphi (x,y)|x\rangle \rangle \\ \;=\frac{1}{16}|3|B\rangle \rangle +\frac{1}{16}|3|B\rangle \rangle +\frac{1}{8}|2|B\rangle +1|G\rangle \rangle \\ \;+\frac{1}{16}|2|B\rangle +1|G\rangle \rangle +\frac{1}{16}|1|B\rangle +2|G\rangle \rangle \\ \;+\frac{1}{8}|2|B\rangle +1|G\rangle \rangle +\frac{1}{16}|2|B\rangle +1|G\rangle \rangle \\ \;+\;\frac{1}{8}|1|B\rangle +2|G\rangle \rangle +\frac{1}{16}|3|G\rangle \rangle +\frac{1}{8}|1|B\rangle +2|G\rangle \rangle \\ \;+\;\frac{1}{16}|1|B\rangle +2|G\rangle \rangle +\frac{1}{16}|1|B\rangle +2|G\rangle \rangle \\ \;=\frac{1}{8}|3|B\rangle \rangle +\frac{3}{8}|2|B\rangle +1|G\rangle \rangle \\ \;+\frac{3}{8}|1|B\rangle +2|G\rangle \rangle +\frac{1}{8}|3|G\rangle \rangle . \end{array}$$

(6)

(4)

The answer to question (a) is thus $\frac{1}{8}$, the probability associated in the last line with the three girls multiset 3|*G*〉.

The interested reader may wish to check that taking the second marginals yields the (expected) age distribution of the form:


$$D(M(\pi_{2}))(\upsilon )=\frac{1}{2}|2|4\rangle +1|5\rangle \rangle +\frac{1}{2}|1|4\rangle +2|5\rangle \rangle .$$

For question (b) we define an observable *g* : *S* → {0, 1} which is 1 if and only if there is at least one girl:


$$\begin{array}{ccccc} g\left(\varphi \right)=1 & \Leftrightarrow & M\left(\pi_{1}\right)\left(\varphi \right)\left(g\right)\geq 1 & \Leftrightarrow & \varphi \left(g,4\right)+\varphi \left(g,5\right)\geq 1. \end{array}$$

This observable *g* is {0, 1}-valued and may be identified with a subset of *S*, as in Lemma 1. Updating υ with *g* involves removing the multisets φ ∈ *S* with *g*(φ) = 0, that is, with boys only, and then renormalising. The normalization factor is the validity:


$$\begin{array}{ccccc} \upsilon ⊧g & = & \underset{\varphi \in S}{\sum }\upsilon \left(\varphi \right)\cdot g\left(\varphi \right) & = & \frac{7}{8} \end{array}$$

The answer to question (b) is obtained by computing the update υ|_*g*_ and taking its children marginal, as before. This yields:


$$\begin{array}{l} D(M(\pi_{1}))(\upsilon {|}_{g})\\ \;=D(M(\pi_{1}))(\frac{1}{7}|1|B,4\rangle +1|B,5\rangle +1|G,4\rangle \rangle \\ \;+\frac{1}{14}|2|B,5\rangle +1|G,4\rangle \rangle +\frac{1}{14}|1|B,5\rangle +2|G,4\rangle \rangle \;\\ \;+\frac{1}{14}|2|B,4\rangle +1|G,5\rangle \rangle +\frac{1}{7}|1|B,4\rangle +1|B,5\rangle +1|G,5\rangle \rangle \;\\ \;+\frac{1}{7}|1|B,4\rangle +1|G,4\rangle +1|G,5\rangle \rangle +\frac{1}{7}|1|B,5\rangle +1|G,4\rangle 1|G,5\rangle \rangle \\ \;+\frac{1}{14}|2|G,4\rangle +1|G,5\rangle \rangle +\;\frac{1}{14}|1|B,4\rangle +2|G,5\rangle \rangle \\ \;+\frac{1}{14}|1|G,4\rangle +2|G,5\rangle \rangle )\\ \;=\frac{3}{7}|2|B\rangle +1|G\rangle \rangle +\frac{3}{7}|1|B\rangle +2|G\rangle \rangle +\frac{1}{7}|3|G\rangle \rangle . \end{array}$$

We can conclude that after seeing one girl the probability that there are three girls has risen from $\frac{1}{8}$ to $\frac{1}{7}$. As an aside: the distribution of age marginals remains the same after this update.

What happens when we see a 4-year old boy? We capture this via an event / observable *b*_4_ : *S* → {0, 1} with *b*_4_(φ) = 1 iff φ(*B*, 4) ≥ 1. Its validity υ ⊧ *b*_4_ in the prior distribution υ is $\frac{7}{12}$. We leave it to the interested reader to verify that the distributions of children / age marginals, after update with *b*_4_, are:


$$\begin{array}{c} D(M(\pi_{1}))(\upsilon {|}_{b_{4}})=\frac{1}{5}|3|B\rangle +\frac{1}{10}|2|B\rangle +1|G\rangle \rangle \\ \;\;\;+\frac{3}{10}|1|B\rangle +2|G\rangle \rangle \\ D(M(\pi_{2}))(\upsilon {|}_{b_{4}})=\frac{3}{5}|2|4\rangle +1|5\rangle \rangle +\frac{2}{5}|1|4\rangle +2|5\rangle \rangle . \end{array}$$

The first equation answers question (c): the probability of three boys is $\frac{1}{5}$, having seen one 4-year old boy. It is higher than the probability of seeing three girls, given that there is at least one girl? The boy-of-4 observation excludes more cases and the remaining cases thus get higher probability, after re-normalization.

The second equation about the age marginals shows that the configuration with two 4-year olds is more likely, after seeing at least one 4-year old (boy). This makes sense.

We can still ask what we can infer if we have seen both a girl and a boy-of-4. As before the order of updating is irrelevant: υ|*g*|*b*_4_ = υ|*b*_4_|*g*. In that situation there are 5 multisets left, out of the original 12, in υ in Equation 10. The distributions of marginals are:


$$\begin{array}{ccc} D(M(\pi_{1}))(\upsilon {|}_{g}{|}_{b_{4}}) & = & \frac{5}{8}|2|B\rangle +1|G\rangle \rangle +\frac{3}{8}|1|B\rangle +2|G\rangle \rangle \\ D(M(\pi_{2}))(\upsilon {|}_{g}{|}_{b_{4}}) & = & \frac{5}{8}|2|4\rangle +1|5\rangle \rangle +\frac{3}{8}|1|4\rangle +2|5\rangle \rangle . \end{array}$$

We conclude by proving in general what we have already seen several times, namely that multiple Bayesian updates commute (Equation 1). We do so by using the conjunction *p & q* (pointwise multiplication) of observables, in order to emphasize the close connection between commutativity of conjunction and of updating. The result below already occurs in Jacobs (2019, Lem. 4.1), together with a generalized formulation of Bayes' rule for observables. A proof is included for completeness.

Proposition 1. Let ω∈D(X) be a distribution with two non-negative observables *p, q* : *X* → ℝ ≥ 0. Then, assuming that the relevant validities are non-zero,


$$\omega {|}_{p}{|}_{q}=\omega {|}_{p\&q}=\omega {|}_{q\&p}=\omega {|}_{q}{|}_{p}.$$

**Proof**. We only have to prove the first equation, since the commutativity of & is obvious (multiplication of numbers is commutative) and the last equation is an instance of the first (with *p, q* swapped). Using the functional description for distributions, we have for *x* ∈ *X*,


$$\begin{array}{l} \omega {|}_{p}{|}_{q}(x)=\frac{\omega {|}_{p}(x)\cdot q(x)}{\omega {|}_{p}⊨q}\\ \;=\frac{\frac{\omega (x)\cdot p(x)}{\omega ⊨p}\cdot q(x)}{\sum_{y}\frac{\omega (y)\cdot p(y)}{\omega ⊨p}\cdot q(y)}\\ \;=\frac{\omega (x)\cdot (p\&q)(x)}{\omega ⊨p\&q}=\omega_{p\&q}(x).\;□ \end{array}$$

(9)

## 5 Concluding remarks

The Bayesian approach is popular in cognition theory, where the human mind is seen as a Bayesian prediction and inference engine, see for instance the recent books (Griffiths et al., 2024; Parr et al., 2022). In that line of work the mismatch caused by the commutativity of Bayesian updating does not get much attention. It is however known in the literature, see notably (Uzan, 2023). One way out is to switch from classical to quantum probability, where conjunction and updating are non-commutative. This has led to a new line of “quantum” cognition theory (see e.g. Busemeyer and Bruza, 2012; Yearsley and Busemeyer, 2016; or Jacobs, 2017b which is similar in style to this article).

When we take the commutativity of Bayesian updating seriously, the proper data structure to deal with multiple updates is: a multiset of observables. Indeed, as we have seen in Section 2, multisets abstract from lists by ignoring the order. This perspective is elaborated in Jacobs (2024), where the different update mechanisms of Pearl and Jeffrey (Jacobs, 2019), and also the variational free update mechanism from predictive coding (Friston, 2009; Tull et al., 2023), are formulated in terms of such multisets of observables. Jeffrey's rule is non-commutative, but in a special way, namely for multiple such (non-singleton) multisets. All this suggests that the topic of commutativity may be a decisive element in further developing probabilistic perspectives in cognition and in AI.

## Data Availability

The original contributions presented in the study are included in the article/supplementary material, further inquiries can be directed to the corresponding author.

## Footnotes

^1^See the special page https://en.wikipedia.org/wiki/Boy_or_girl_paradox.

^2^One can obtain the distribution υ in Equation 10 itself via conditioning, namely from the multinomial distribution, of draws of size three from the uniform distribution on *C*×*A*. One updates this multinomial distribution by keeping only those multiset φ in which both ages occur, that is, for which the support $supp\left(M\left(\pi_{2}\right)\left(\varphi \right)\right)\subseteq A$ of the second marginal of φ has two elements. This construction of υ distracts from the main line, so we decided to simply present the relevant prior distribution in Equation 10.
