# Supplementary material for: Commutativity of probabilistic belief revision
Source: Front Cognit. 2025 Aug 6;4:1623227. doi: 10.3389/fcogn.2025.1623227 (PMC13271130; doi:10.3389/fcogn.2025.1623227)
Supplement: Supplementary file 1 [file Supplementary_file_1.pdf]

Appendix

This appendix assumes familiariy with category theory [2, 27, 32, 34, 5, 31]., especially in relation to probability theory. For more information, see [29, 9, 20]. The duality theory that we sketch below is elaborated in greater detail (for non-negative measurable observables) in [4].

A Kadison duality

There are famous duality results in mathematics, see *e.g.* [25], for instance the one of Stone, between the category **CH** of compact Hausdorff spaces and the opposite $\mathbf{BA}^{\mathrm{op}}$ of the category of Boolean algebras, connecting 'spatial' and 'logical' perspectives, see [25]. There is also Gelfand duality between **CH** and the opposite of the category of commutative unital C^*-algebras. Here we use Kadinson duality between the category $\mathbf{CCH}_{\mathrm{ps}}$ of convex compact Hausedorff spaces (with a certain point separtation property) and the opposite $\mathbf{BOUS}^{\mathrm{op}}$ of the category of Banach order unit spaces, see [1] and [7, Thm. 11.3]. The proof of this duality is very complex and will be skipped here. We generally follow the presentation of [21].

Theorem 2 (Kadison)**.** *There is a duality (equivalence of categories) via 'homming into* $\mathbb{R}$*', of the form:*

$$\mathbf{BOUS}^{\mathrm{op}} \underset{AC(-,\mathbb{R})}{\overset{LPU(-,\mathbb{R})}{\rightleftarrows}} \mathbf{CCH}_{\mathrm{ps}} \qquad (\simeq)$$

The abbreviations LPU and AC stand for 'Linear Positive Unital' and 'Affine Continuous'. They will be explained below.

We review the essentials of this duality and refer to [21] for further details.

- An *order unit space* is a partially ordered vector space V over $\mathbb{R}$ with a strong unit $1 \in V$. The latter is a positive element $1 > 0$ such that for each $v \in V$ there is an $n \in \mathbb{N}$ with $v \leq n \cdot 1 = 1 + \cdots + 1$, n times. This unit is called Archimedean: if $v \leq r \cdot 1$ for all positive $r \in \mathbb{R}$, implies $v \leq 0$. This property makes it possible to define a norm $\|v\| = \bigwedge\{r \geq 0 \mid -r \cdot 1 \leq v \leq r \cdot 1\}$. A Banach order unit space is then an order unit space whose unit is Archimedean and whose induced distance (via the norm) makes the space metrically complete.

  A morphism $f \colon V \to W$ in the category **BOUS** is a linear function that is positive — $v \geq 0$ implies $f(v) \geq 0$, or equivalently, $v \geq v'$ implies $f(v) \geq f(v')$ — and preserves the unit: $f(1) = 1$.

- A set A is called convex if it is closed under convex sums: for finitely many $a_i \in A$ and $r_i \in [0, 1]$ with $\sum_i r_i = 1$, one has $\sum_i r_i \cdot a_i \in A$. A function

is called affine if it preserves such convex sums. We write **CCH** for the category of convex compact Hausdorff spaces, with affine continuous maps between them. The subcategory $\mathbf{CCH}_{\mathrm{ps}} \hookrightarrow \mathbf{CCH}$ contains those $A \in \mathbf{CCH}$ with a point separation property: if $a \neq a'$, then there is an affine continuous map $f\colon A \to [0,1]$ with $f(a) \neq f(a')$.

When X is a finite set, the set $Obs(X) = \mathbb{R}^X$ of observables on X, from Definition 3 (1), is a Banach order unit space. Also, the set $\mathcal{D}(X)$ is a convex compact Hausdorff space. It separates points, since if $\omega, \omega' \in \mathcal{D}(X)$ are unequal, they must satisfy $\omega(a) \neq \omega'(a)$ for some $a \in X$. The evaluation map $ev_a\colon \mathcal{D}(X) \to [0,1]$ sending $\rho \in \mathcal{D}(X)$ to $ev_a(\rho) = \rho(a)$ is both continuous (since Lipschitz) and affine. Obviously, $ev_a(\omega) \neq ev_a(\omega')$.

We write $\mathcal{K}\ell_f(\mathcal{D}) \hookrightarrow \mathcal{K}\ell(\mathcal{D})$ for the Kleisli category of the distribution monad $\mathcal{D}$ restricted to finite sets. The next result relates probabilistic computation (between finite sets) in this category $\mathcal{K}\ell_f(\mathcal{D})$ to Kadison duality.

Theorem 3. *There are observation and states functors in a triangle that commutes up-to-isomorphism of the form:*

$$\begin{array}{ccc} \mathbf{BOUS}^{\mathrm{op}} & \simeq & \mathbf{CCH}_{\mathrm{ps}} \\ {\scriptstyle Obs}\nwarrow & & \nearrow{\scriptstyle Stat} \\ & \mathcal{K}\ell_f(\mathcal{D}) & \end{array} \tag{11}$$

where $Obs(X) = \mathbb{R}^X$ *and* $Stat(X) = \mathcal{D}(X)$. *For a Kleisli map* $c\colon X \to \mathcal{D}(Y)$ *there are maps:*

$$\begin{array}{ccc} Obs(Y) \xrightarrow{c^* = Obs(c)} Obs(X) & \qquad & Stat(X) \xrightarrow{c_* = Stat(c)} Stat(Y) \\ q \longmapsto \Big(x \mapsto \sum_{y \in Y} c(x)(y) \cdot q(y)\Big) & & \omega \longmapsto \Big(y \mapsto \sum_{x \in X} \omega(x) \cdot c(x)(y)\Big) \end{array} \tag{12}$$

These operations c^* *and* c_* *are called observation transformation and state transformation, working in different directions. The two upgoing functors Obs and Stat in the triangle* (11) *are both full and faithful, so that there are close relations between maps in any of the three categories in the triangle, that is between computations, observable transformations, and state transformations, see [15] for more information.*

The above triangle (11) *commutes (up-to isomorphism) via two isomorphisms of the form:*

$$LPU\big(Obs(X), \mathbb{R}\big) \xrightarrow[\cong]{\alpha} \mathcal{D}(X) \qquad\qquad AC\big(\mathcal{D}(X), \mathbb{R}\big) \xrightarrow[\cong]{\beta} Obs(X) \tag{13}$$

Functions $Obs(X) \to \mathbb{R}$, *which are linear, positive and unit-preserving, will be called* belief functions. *They correspond to distributions, via the above isomorphism* α.

Proof. It is not hard to see that the observable transformation $c^* \colon \mathit{Obs}(Y) \to \mathit{Obs}(X)$ is linear and positive and preserves the unit, and that the state transformation $c_* \colon \mathcal{D}(X) \to \mathcal{D}(Y)$ is affine and continuous (since it is Lipschitz). We will first show that the triangle (11) commutes up to isomorphism.

For a finite set X there is the isomorphism on the left of (13):

$$\mathit{LPU}\big(\mathbb{R}^X, \mathbb{R}\big) \xrightarrow[\cong]{\alpha} \mathcal{D}(X) \qquad \text{given by:} \qquad \alpha(h) \coloneqq \sum_{x \in X} h(\mathbf{1}_x)\big|\, x\,\big\rangle, \tag{14}$$

where $\mathbf{1}_x = \mathbf{1}_{\{x\}} \colon X \to \{0,1\} \hookrightarrow \mathbb{R}$ is the point observable given by $\mathbf{1}_x(x') = 1$ when $x = x'$ and $\mathbf{1}_x(x') = 0$ when $x \neq x'$. This $\alpha(h)$ is indeed a distribution since h is linear and preserves the unit: $\sum_x \alpha(h)(x) = \sum_x h(\mathbf{1}_x) = h\big(\sum_x \mathbf{1}_x\big) = h(\mathbf{1}) = 1$. The inverse is given by validity $\alpha^{-1}(\omega)(p) = \omega \models p$.

Next, there is also the isomorphism on the right in (13):

$$\mathit{AC}\big(\mathcal{D}(X), \mathbb{R}\big) \xrightarrow[\cong]{\beta} \mathbb{R}^X \qquad \text{where} \qquad \beta(f)(x) \coloneqq f\big(1|\, x\,\rangle\big). \tag{15}$$

This works since each affine map $\mathcal{D}(X) \to \mathbb{R}$ is automatically Lipschitz, and since $\mathcal{D}(X)$ is the free convex set on X, so that affine maps $\mathcal{D}(X) \to \mathbb{R}$ correspond to observables $X \to \mathbb{R}$.

Finally we show that the functor $\mathit{Obs} \colon \mathcal{K}\ell_f(\mathcal{D}) \to \mathbf{BOUS}^{\mathrm{op}}$ is full and faitful. Via the isomorphisms in (14) one can show that the functor $\mathit{Stat} \colon \mathcal{K}\ell_f(\mathcal{D}) \to \mathbf{CCH}_{\mathrm{ps}}$ is then also full and faitful.

- First, for faithfulness, let $c, d \colon X \to \mathcal{D}(Y)$ satisfy $c^* = d^* \colon \mathit{Obs}(Y) \to \mathit{Obs}(X)$. Then, using point observables, as above, for each $x \in X$ and $y \in Y$, $c(x)(y) = c^*(\mathbf{1}_y)(x) = d^*(\mathbf{1}_y)(x) = d(x)(y)$.

- For fulness, let $h \colon \mathit{Obs}(Y) \to \mathit{Obs}(X)$ be a linear and positive map that preserves the unit. We define $c \colon X \to \mathcal{D}(Y)$ as $c(x)(y) = h(\mathbf{1}_y)(x)$. This c is a well-defined Kleisli map since each $c(x)$ is a distribution, with probabilities adding up to one:

$$\begin{aligned}\sum_{y \in Y} c(x)(y) \;=\; \sum_{y \in Y} h(\mathbf{1}_y)(x) \;&=\; \left(\sum_{y \in Y} h(\mathbf{1}_y)\right)(x) \\ &=\; h\left(\sum_{y \in Y} \mathbf{1}_y\right)(x) \;=\; h(\mathbf{1})(x) \;=\; \mathbf{1}(x) \;=\; 1.\end{aligned}$$

  Also, $\mathit{Obs}(c) = c^*$ is the original map h since we can write an arbitrary observable $q \in \mathit{Obs}(Y)$ as sum $q = \sum_y q(y) \cdot \mathbf{1}_y$, using that Y is finite.

Thus:

$$\begin{aligned} h(q)(x) &= h\left(\sum_{y\in Y} q(y)\cdot \mathbf{1}_y\right)(x) \\ &= \left(\sum_{y\in Y} q(y)\cdot h(\mathbf{1}_y)\right)(x) \\ &= \sum_{y\in Y} q(y)\cdot h(\mathbf{1}_y)(x) \;=\; \sum_{y\in Y} q(y)\cdot c(x)(y) \;=\; c^*(q)(x). \qquad \square \end{aligned}$$

The above triangle in (11) is a mathematically perfect situation for program semantics, where programs are represented as maps c in the Kleisli category at the bottom. These maps bijectively correspond to two transformer operations, c^* between observations / predicates (backward) and c_* between distributions / states (forward). This backward transformation of observables yields what is called the weakest precondition of a program. The forward transformation of distributions captures how running a program in a particular state yields a successor state. Within a quantum setting these backward transformers are associated with Heisenberg's approach and the forward transformers with Schrödinger's approach, see [15] for details. In the next section we associate the left-hand (Heisenberg) side in the triangle (11) with Bayesian statistics and the right-hand (Schrödinger) side with frequentist statistics.

B Updating across duality

In Definition 4 we have introduced the updated distribution $\omega|_p$ via a concrete formulation (9). The goal of this section is to reconstruct this formulation via the duality of the previous section.

There is the famous rule of Bayes. For events E, D one may use the conditional probability $P(E \mid D)$ of E given D. It satisfies the equations:

$$\frac{P(E \,\&\, D)}{P(D)} = P(E \mid D) = \frac{P(D \mid E)\cdot P(D)}{P(D)}. \tag{16}$$

The first equation is sometimes called the product rule that defines $P(E \mid D)$; it was derived in Lemma 1. The second equation is Bayes' rule. Here we have to assume that the probability $P(D)$ of the event D on which we condition is non-zero.

In this section we show that via the isomorphism (14) one can also formulate updating for distributions, on the frequentist side. For some reason this is not done in the traditional literature on probability theory. It is however a useful approach, as we illustrate below.

We fix a distribution $\omega \in \mathcal{D}(X)$, for a finite set X, together with a non-negative observable $p \in \mathit{Obs}(X)$, thus satisfying $p \geq \mathbf{0}$. We assume that the

validity $\omega \models p$ is non-zero. We write validity for expected value, see Definition 3 (3). Consider the belief function $h\colon Obs(X) \to \mathbb{R}$ given as:

$$h(q) \;=\; \frac{\omega \models p \,\&\, q}{\omega \models p} \qquad \text{that is} \qquad h \;=\; \frac{\omega \models p \,\&\, (-)}{\omega \models p}. \tag{17}$$

Recall that we write $p \,\&\, q$ for the pointwise multiplication of observables. It is not hard to see that h is linear and positive (since $p \geq \mathbf{0}$) and sends the unit observable $\mathbf{1}$ to 1. This function h is the analogue for observables of the leftmost expression in (16), defining conditional probability, but now generalised to observables. The crucial step is to apply the isomorphism (14) to this function h, in order to get a form of updating for distributions, in the world of frequentism.

Theorem 4. *Consider a random variable* $\big(\omega \in \mathcal{D}(X), p\colon X \to \mathbb{R}\big)$ *where* X *is finite,* $p \geq \mathbf{0}$ *and* $\omega \models p$ *is non-zero. Write* $\omega|_p \in \mathcal{D}(X)$ *for the distribution obtained via the isomorphism* $\alpha\colon LPU\big(Obs(X), \mathbb{R}\big) \xrightarrow{\cong} \mathcal{D}(X)$ *in* (13)*, applied to the belief function in* (17)*.*

$$\omega|_p \;\coloneqq\; \alpha\left(\frac{\omega \models p \,\&\, (-)}{\omega \models p}\right).$$

This updated distribution $\omega|_p$ *satisfies the following properties.*

1. *This distribution* $\omega|_p$ *obtained in this abstract manner satisfies the concrete description that was used earlier in* (9)*:*

$$\omega|_p \;=\; \sum_{x \in X} \frac{\omega(x) \cdot p(x)}{\omega \models p} \,\big|\, x \big\rangle. \tag{18}$$

2. *The analogue of* (16) *then holds: for arbitrary* $q \in Obs(X)$*,*

$$\frac{\omega \models p \,\&\, q}{\omega \models p} \;=\; \omega|_p \models q \;=\; \frac{(\omega|_q \models p) \cdot (\omega \models q)}{\omega \models p}. \tag{19}$$

Proof. 1. Following the definition of α in (14) gives the formulation in (9):

$$\begin{aligned}
\omega|_p \;=\; \alpha\left(\frac{\omega \models p \,\&\, (-)}{\omega \models p}\right) &= \sum_{x \in X} \frac{\omega \models p \,\&\, \mathbf{1}_x}{\omega \models p} \,\big|\, x \big\rangle \\
&= \sum_{x \in X} \frac{\sum_y \omega(y) \cdot p(y) \cdot \mathbf{1}_x(y)}{\omega \models p} \,\big|\, x \big\rangle \\
&= \sum_{x \in X} \frac{\omega(x) \cdot p(x)}{\omega \models p} \,\big|\, x \big\rangle.
\end{aligned}$$

2. The first equation in (19) is obtained via the inverse of α, given by validity, as described in the proof of Theorem 3.

$$\omega|_p \models q \;=\; \alpha^{-1}\big(\omega|_p\big)(q) \;=\; \alpha^{-1}\left(\alpha\left(\frac{\omega \models p \,\&\, (-)}{\omega \models p}\right)\right)(q) \;=\; \frac{\omega \models p \,\&\, q}{\omega \models p}.$$

The second equation in (19) now follows by using the first equation with p and q exchanged — and the commutativity of $\&$. □
